# Blood transcriptomics and metabolomics for personalized medicine

**DOI:** 10.1016/j.csbj.2015.10.005

**Published:** 2015-10-31

**Authors:** Shuzhao Li, Andrei Todor, Ruiyan Luo

**Affiliations:** a Department of Medicine, Emory University School of Medicine, 615 Michael Street, Atlanta, GA 30322, USA; b Division of Epidemiology and Biostatistics, School of Public Health, Georgia State University, One Park Place, Atlanta, GA 30303, USA

**Keywords:** Transcriptomics, Metabolomics, Blood systems biology, Personalized medicine, Data integration

## Abstract

Molecular analysis of blood samples is pivotal to clinical diagnosis and has been intensively investigated since the rise of systems biology. Recent developments have opened new opportunities to utilize transcriptomics and metabolomics for personalized and precision medicine. Efforts from human immunology have infused into this area exquisite characterizations of subpopulations of blood cells. It is now possible to infer from blood transcriptomics, with fine accuracy, the contribution of immune activation and of cell subpopulations. In parallel, high-resolution mass spectrometry has brought revolutionary analytical capability, detecting > 10,000 metabolites, together with environmental exposure, dietary intake, microbial activity, and pharmaceutical drugs. Thus, the re-examination of blood chemicals by metabolomics is in order. Transcriptomics and metabolomics can be integrated to provide a more comprehensive understanding of the human biological states. We will review these new data and methods and discuss how they can contribute to personalized medicine.

Many human diseases are complex and heterogeneous, whereas diagnostic methods are still limiting. Genetics and high-throughput molecular profiling now helps to redefine the disease classifications [1], [2]. Personalized and precision medicine aims to design therapeutic interventions based on the condition of individual patients. For example, in the case of *trastuzumab*, a drug that is administered to breast cancer patients, its therapeutic efficiency varies depending on the patient’s breast cancer subtype. This is because *trastuzumab* targets HER2 (human epidermal growth factor receptor type 2) proteins, and it is only effective on breast cancers with HER2 overexpression [3]. Therefore, a diagnostic test that determines HER2 overexpression is required before *trastuzumab* can be subscribed. A different type of example is adoptive T cell transfer for cancer immunotherapy, where specific T cells from an individual patient are engineered and expanded, then infused back to the same patient [4], [5], [6]. This type of therapy is “double” personalized because the T cells have to be from the very patient to be immunologically tolerant, and their surface receptors have to be specific to the tumor mutation found in that patient. Numerous examples exist that drug efficacy is limited due to the lack of “precision” mechanism. The widely used statins (cholesterol lowering drugs) may be efficacious in only 5% of the population, while esomeprazole (for heartburn treatment) fares even less [7]. A lot of research efforts have gone to identifying genetic variations associated with diseases, including many large genome-wide association studies (GWAS). However, genetic variations only account for small percentages of the occurrence of common diseases [8], [9]. It is increasingly recognized that there is a large gap between genomics and phenotypes and that transcriptomics and metabolomics are important to fill this gap [10], [11], [12], [13], [14]. In this article, we will review the latest progress in transcriptomics and metabolomics, with a focus on samples from blood, a key tissue for clinical diagnosis. Since abundant introductory literature can be found on omics technologies and their data analysis, this article focuses more on important recent developments and opportunities.

## 1 An overdue review of “blood systems biology”

**Fig. 1:**
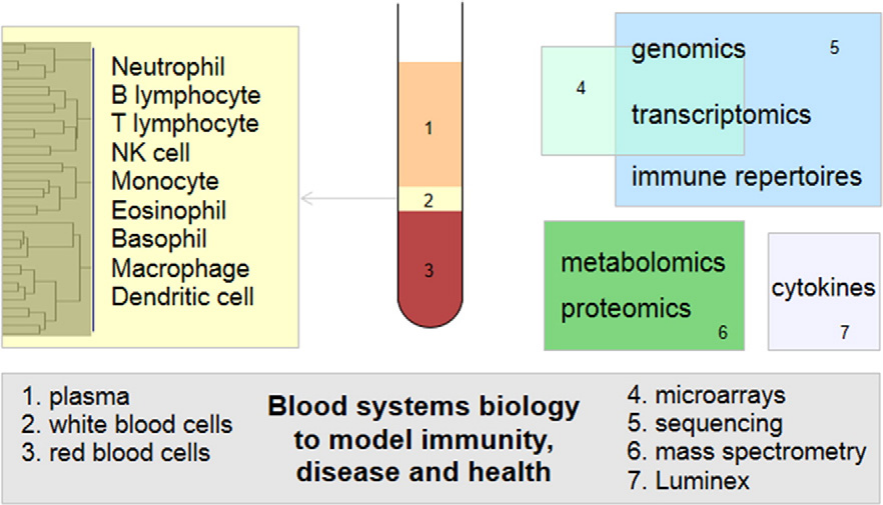
Overview of blood systems biology, the pertinent samples and technologies. After a blood sample is taken, it is easily separated into plasma, white blood cells and red blood cells. The major white blood cells are listed on the left, while each cell type can be analyzed via exquisite protein markers via flow cytometry, giving information on particular subpopulations. Major “omics” technologies are listed on the right. DNA microarrays overlap with both genomics (genotyping arrays) and transcriptomics (expression arrays). DNA sequencing supports genomics (and epigenomics), transcriptomics (RNAseq), and immune repertoires. Immune repertoires include T cell receptor and B cell receptor sequences, whereas the latter represents antibody diversity. Both metabolomics (and environmental chemical exposures) and proteomics are largely dependent on mass spectrometry.

Blood has been intensively investigated since the beginning of molecular systems biology. Publications on disease diagnosis using blood transcriptomes are now numbered in thousands. Although it is widely recognized that mRNA only provides a slice of information from complex biology, few papers attempted to quantify the cell-level complexity in blood transcriptomics. Because blood is a mixture of many different cell types (Fig. 1), the fluctuation of cell populations alone causes large variations in transcriptomics data. This problem only became tractable with the recent progress in human immunology, where transcriptomics of isolated cell populations provided necessary information [15], [16], [17]. Nonetheless, a review on “blood systems biology” is long overdue.

As part of the body circulatory system, blood reflects the homeostasis of metabolism, hematopoietic development, and immune functions. As Fig. 1 shows, this involves many cell types and subtypes, and a number of “omics” technologies are employed to measure on different aspects of the system. The global molecular profiles of different cell types are tightly related to their developmental lineage and functions. As Novershtern et al. [18] showed, the clustering of transcriptomics data of blood cells reflects the hematopoietic process. The white blood cells are also sensitive indicators of the immune status. An infection will readily induce the influx of immune cells to blood as well as the activation of molecular programs in these cells. Cytokines and chemokines can increase dramatically during such events. The plasma contains molecular signals and wastes from the lymphatic system. The metabolites within plasma can reflect liver or kidney function, endocrine signaling, inflammation, and metabolic disorders. Thus, blood systems biology needs to address the following: (1) mixture data—most commonly, omics data are collected on peripheral blood mononuclear cells, where cell population composition is critical; (2) connection to a systemic model, such as pharmacokinetics or host-pathogen interaction models—blood is not a closed system by itself, only a window to systemic events; and (3) data integration. This could be the association between omics data and phenotype or the connection between different omics data types. We will start with an overview of transcriptomics and metabolomics then move on to specific topics for “blood systems biology”.

## 2 Data acquisition of transcriptomics and metabolomics

DNA microarrays were developed in the 1990s as a major technology to measure transcriptomics. The technology relies on the specific hybridization between complementary polynucleotides. Probes are designed based on known gene transcripts and tethered on a glass surface. Targets are generated from biological samples, labeled directly or indirectly with fluorescent dyes. The hybridization reactions are carried on in miniaturized chambers. After the probes capture specific targets, the fluorescent signals are scanned and reported based on their grid locations. Thousands of microarray experiments are now deposited in public repositories such as GEO [19] and ArrayExpress [20].

As the cost of DNA sequencing drops, RNAseq becomes a viable alternative to capture transcriptomics. Using massively parallel sequencing platforms, RNAseq reads the number of DNA copies that are converted from mRNA, thus quantifying the concentration of mRNA species. From these sequencing reactions, the sequence variations in exons, such as single nucleotide polymorphisms (SNPs) and alternative splicing, are also captured in the data. Both the experimental methods and the computational analysis of RNAseq are evolving rapidly, and significant improvements are expected.

Metabolomics is the global profiling of small molecules (usually under 2000 Da). While nuclear magnetic resonance (NMR) [21] has been a powerful tool, mass spectrometry coupled with liquid or gas chromatography is the most popular platform due to the superior sensitivity and coverage [22], [23], [24]. The newest high-resolution mass spectrometer, in particular, yields unparalleled precision in analyzing chemicals in complex biological samples. The basic principle used by mass spectrometers is the differentiated deflection of charged particles in a magnetic field based on their mass. By the Lorentz law, the magnitude of the deflection is proportional to the mass to charge ratio. The advanced version, Fourier transform mass spectrometers, can achieve spectacular mass resolution by measuring the spinning frequency of ions that are trapped and oscillate in a chamber. The computational aspects of metabolomics are also in rapid progress, including open source feature extraction tools (XCMS [25], OpenMS [26], apLCMS [27], xMSanalyzer [28]), databases of metabolites (Human Metabolome Database [29], [30], METLIN [31], PubChem [32], ChEBI [33]), and data analysis tools (XCMS Online [34], MetaboAnalyst [35], mummichog [36]). It should be noted that these data contain more than endogenous metabolites, also including food intake, microbial activities, pharmaceutical drugs, and environmental exposures. The collective measurement is sometimes termed as “exposome” [37], [38].

For the analysis of both transcriptomics and metabolomics data, general principles of “omics” apply. With measurement of thousands of features, multiple test correction is necessary to control false positive rates [39], [40]. The number of features is usually far larger than the number of samples. Statistical methods often “borrow” information from variation between features to help the estimation of variation between samples [41]. Prior knowledge of molecular pathways and interactions can be of great value, and the methods usually involve over-representation tests or network modeling [42], [43]. Since these areas can be referred to other more generic reviews, we will highlight a few areas that are more pertinent to blood data: how to deal with data from the mixture of blood cells, emerging metabolomics data on plasma or serum, and useful pathway and network tools.

## 3 Untangling mixed cell populations in blood transcriptomics

When transcriptomics data are measured on a mixture of multiple cell populations, it is a reasonable assumption that the data are a linear combination of transcriptomes of each populations [44]. These separate cell populations can be obtained by flow cytometry-based sorting, and large quantity of data are made available in ImmGen and ImmPort databases [15], [16]. Conversely, if the percentages of each cell population are known, variations may be attributed to each population by regression methods [45].

**Fig. 2:**
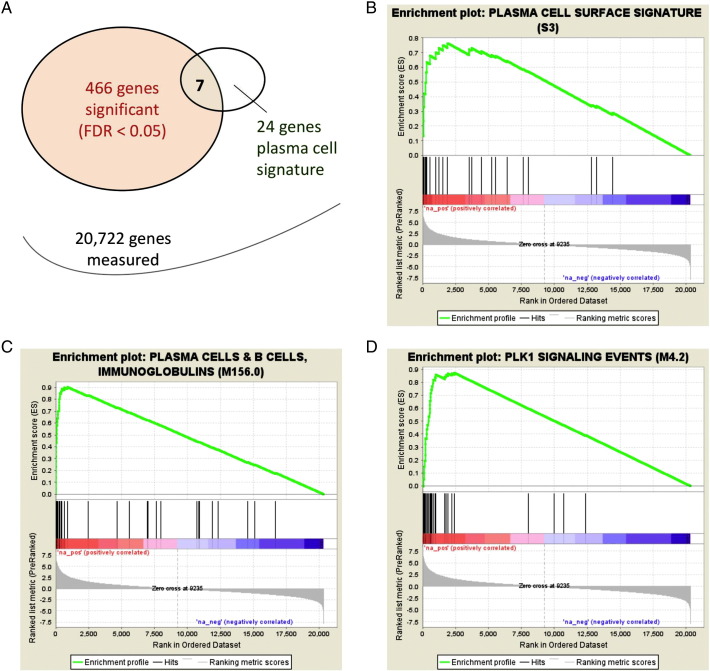
Testing cell populations and gene modules in blood transcriptomics. This demonstration is based on a paired comparison between day 7 and baseline in MCV4 vaccination [46]. Common statistical methods for pathway analysis are used here, while we replace conventional pathways with cell-specific signatures or custom gene modules. (A) Over-representation test. DNA microarray data are collapsed to the gene level by using the probe set of highest intensity per gene. Gene expression values are compared by paired *t*-test, and corrected for false discovery rate [50]. Among the significant genes identified here, 7 are found in a predefined signature of plasma cells. These numbers are used to construct a contingency table, and Fisher exact test returns an enrichment *p*-value < 1E^− 5^. (B) The distribution of the same plasma cell signature genes is tested by GSEA. The bottom color bar shows the distribution of all genes, ranked by t-score between two time points. The vertical lines indicate the positions of the 24 genes on the ranked list, which are highly skewed for upregulation. (C) A gene module from the BTM collection [46] provides better measurement of antibody secreting cells, demonstrated on the same data. (D) Additional example of BTM module on PLK1 signaling, showing highly significant enrichment towards upregulation. The *p*-values in B, C, and D approach zero. A detailed tutorial on BTMs is available as an online supplement to Li et al. [46].

Since “omics” data are often noisy, pre-filtered cell-type-specific genes (markers) are very useful in this context [46], [47], [48]. The use of too few markers, like those in flow cytometry, is not recommended in transcriptomics analysis because a larger number of genes are needed to counter the measurement noise, and time differential may exist between protein (used in flow cytometry) and mRNA (measured in transcriptomics) levels. A set of cell-type-specific genes are included in the blood transcription modules from Li et al. [46]. With cell-type-specific markers, a statistical test of over-representation can reveal what cell type contributes to the most differential genes [42], [48]. An example is shown in Fig. 2A: immunization using MCV4 vaccine upregulated 466 significant genes after 1 week. These genes contain 7 out of 24 signature genes for plasma cells, the major antibody secreting cells. Given that these numbers were drawn from genome-wide measurement of 20,722 genes, the enrichment on plasma cell signature genes is highly significant (*p* < 10^− 5^, Fisher exact test). Alternatively, one can leverage the GSEA (Gene set enrichment analysis [49]) statistical framework, using cell-specific markers as gene sets. This method shows that the same plasma signature of 24 genes are highly enriched for upregulated genes (*p*-value approaching 0, Fig. 2B). The GSEA approach can be more sensitive than over-representation tests and less biased by cutoffs in feature selection. In general, we have found that distribution tests in the style of Kolmogorov–Smirnov test suit well for assigning cell type information from blood transcriptomics, and the results are very consistent with flow cytometry data that were obtained on the same samples (unpublished).

## 4 Metabolomics for disease markers

While transcriptomics analysis usually requires cell collection protocols in place to preserve the integrity of mRNA, metabolomics is amendable to most archival samples. This easy access to samples and the reasoning that metabolites provide functional readout of gene activities gather a great deal of enthusiasm to look for disease markers using metabolomics [51], [52], [53], [54], [55], [56], [57], [58], [59], [60], [61], [62], [63], [64], [65]. Examples of metabolomics for biomarker study include diabetes [62], [66], macular degeneration [67], asthma [68], Parkinson’s disease [69], nonalcoholic fatty liver disease [70], and tuberculosis [71]. Notably, metabolite markers of diabetes were reported many years prior to the disease onset [61]. The field of high-resolution metabolomics is advancing very rapidly [24], [72]. Although it has been difficult to compare earlier data from different platforms, the accumulation of high-resolution metabolomics data may be approaching a critical threshold of assembling a reference human metabolome.

**Fig. 3:**
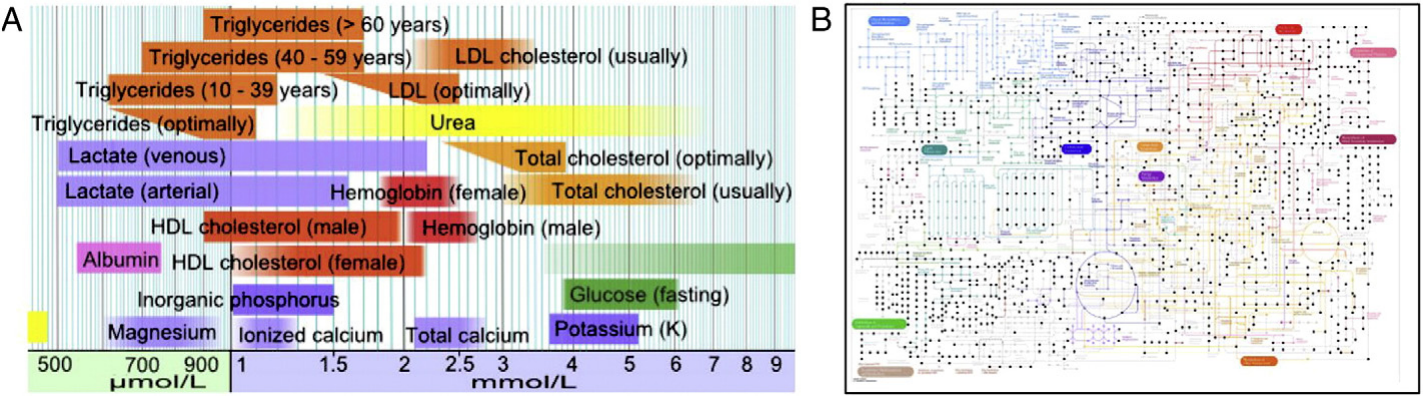
Metabolomics as potential alternative to clinical blood test. (A) Partial chart of chemicals in blood test (adopted from [74]). The physiological ranges of several metabolites are shown by log scale. (B) Current coverage on KEGG pathways by LC-MS metabolomics, using data generated from our group. Each black dot is a matched metabolite. The full KEGG metabolic map can be viewed at high resolution at http://www.genome.jp/kegg/pathway/map/map01100.html. As metabolomics technology progresses, it can be expected to quantify over 1000 chemicals in less than 10 min. Such data will be able to support a much more detailed diagnostic chart.

The current clinical blood tests report a limited number of metabolites (Fig. 3), most of which are detected in current metabolomics data. That is, with similar cost, metabolomics can already deliver quantitative information on hundreds of metabolites. The normal and abnormal ranges of many metabolites are either already in the literature or can be learned from large cohorts. Recently, Miller et al. [73] have already demonstrated that a single metabolomic analysis successfully diagnosed 20 inborn metabolic diseases. The potential of clinical metabolomics is revolutionary—once proofs of new disease markers sink in and regulatory approval comes, metabolomics can become a powerful tool for universal health screen.

## 5 Pathways and modules—power in groups

While statistical analysis of “omics” data is often penalized by false discovery rates, pathway analysis is powerful because it both brings in the context of prior knowledge and increases the statistical power while doing so [42], [43]. However, the curation of pathways contains inherent human bias and is sometimes incomplete, i.e., genes of consequence are missing. In fact, pathway analysis has severe limitations when it comes to the complex data of blood transcriptomics. First, the current pathway databases are biased towards cancer, under-representing the immunology in white blood cells. Second, many pathways are based on tissues other than blood. Third, pathways poorly capture signaling cross-talks and intercellular communications. Fourth, genes in a sequential pathway may be expressed at different time, which is easily masked by heterogeneous populations of cells. Moreover, many pathways were discovered under extreme perturbations that do not reflect physiological conditions. Finally, the important context of cell types is usually missing in pathway databases.

To amend these above issues, Li et al. [46] undertook a large-scale integration of transcriptomics to define detailed molecular mechanisms in human antibody response. Using blood transcriptomics data from over 500 public studies, high-quality gene networks were reverse engineered via a mutual information approach. The resulting blood transcription modules (BTM) were validated by prior knowledge, as they recovered known protein complexes and recaptured immunological events in the literature. They also demonstrated superior sensitivity over canonical pathways. Using this new toolset of BTMs, distinct antibody response programs were identified for different types of vaccines. Examples of using BTMs as alternative to canonical pathways are shown in Fig. 2C and D, in combination with the popular GSEA software. Other efforts along this direction include a modular framework of blood genomics [75] and common axes of peripheral blood gene expression [76]. Better database curation is also under the way [49], [77] (Godec et al., submitted).

The power of pathways and modules is also sought by computational metabolomics. Xia and Wishart [78] developed a metabolite set enrichment analysis, where metabolite modules were based on prior human curation. Deo et al. [79] built data-driven modules and identified a significant group of transporter reactions that escaped previous pathway curation. Li et al. [36] took the concept of metabolic pathways and networks to high-throughput metabolomics data without prior annotation. They used the collective statistical power in metabolic knowledge to resolve the ambiguity in computational prediction of metabolite identity, therefore predicting pathway and module activity in one step. This method, under the name of *mummichog*, becomes a powerful tool to accelerate metabolomics studies [80], [81], [82].

## 6 Integrating different data types to understand disease pathophysiology

The analysis of “omics” data is challenging and has motivated many new developments in informatics and statistics. However, each “omics” experiment only captures a static picture of dynamic and complex biology and often an averaged value of mixed signals, e.g., from many heterogeneous cells. The integration of different data types will result in a more complete understanding of disease pathophysiology and combine experimental evidences to filter out noisy signals [83], [84], [85].

Data integration can be a knowledge-driven process. For instance, enzyme proteins connect metabolites by catalyzing their conversions, and such knowledge is collected in metabolic models and databases (e.g., KEGG [86], BioCyc [87], and Reactome [88]). Guo et al. [89] recently reported that the integration of metabolomics and genomics, by matching metabolite concentration to genetic mutation on the corresponding enzymes, was successful to explain several physiological abnormalities and disease risks in relatively healthy volunteers. Genes and proteins are often conveniently organized into the annotation of genomes. In the absence of prior curation, data-driven processes become necessary. For instance, transcriptomics data can be associated with genomic QTLs (quantitative trait loci) and denoted as expression QTL or “eQTL” [14], [90]. Similarly, metabolomics data can support the notion of metabolomic QTL, “mQTL” [91], [92].

Real-world data are often heterogeneous and require the combination of multiple methods. For example, the analysis tool for heritable and environmental network associations (ATHENA) [93] was developed to examine the associations between copy number alterations, methylation, microRNA, and gene expression with ovarian cancer survival. A neural network model was constructed for each data type separately, and the variables from the best models of each individual data set were then combined to create an integrative model using grammatical evolution neural networks (GENN) and grammatical evolution symbolic regression [94], [95]. The statistical methods in ATHENA include symbolic regression, artificial neural networks, support vector machines, and GENN. These methods are selected based on a number of criteria, including fitting accuracy and robustness to non-linear interactions. Bayesian networks are also incorporated to identify conditional relationships.

Bayesian networks (BN) are a flexible and powerful method in integrating multiple “omics” data and prior information [96], [97], [98], [99], [100], [101]. BNs are directed acyclic graphs in which the edges of the graph describe the conditional dependencies (given information on parent nodes) between nodes and nodes are random variables representing quantitative traits such as expression levels of genes, proteins, or metabolites. The unconnected nodes in the network represent the genes or metabolites that are conditionally independent of each other, given the parent information. Information from known interactions and pathways can be used to generate prior information of graph structure. Different weights (prior probabilities) can be given to nodes or edges reflecting researchers’ belief of the structure. Even though edges in BNs are directed, they do not represent causal relationships. However, the BN reconstruction algorithm can infer causal directions in the network by taking additional information as priors. For example, genes with cis-eQTLs (*cis* means locally acting on a genomic sequence) could be parent nodes of genes with coincident trans-eQTLs (*trans* means distally acting), but genes with trans-eQTLs are not allowed to be parents of genes with cis-eQTLs; information flows from DNA to mRNA but not in the reverse direction.

## 7 Concluding remarks

In the gap of common diseases and genomics, transcriptomics and metabolomics provide the important functional link and thus are key components to guide the development of personalized precision medicine. Rapid progress has been made in both areas very recently. Blood transcriptomics has now absorbed many details of human immunology. The example of blood transcription modules [46] is a powerful tool to gauge systemic immune response from blood transcriptomics, capturing changes in both cell populations and immune pathways in general populations. Metabolomics is a fast-growing technology that captures both endogenous metabolites and environmental exposures. These data overlap with blood tests performed by current clinical methods but offer a much more powerful future alternative. The advent of these capabilities impacts many scientific and biomedical fields.

By definition, personalized medicine is an “*n* = 1” problem, which however, does not mean there is less biological complexity in a single person. For that very reason, in the past few decades, the translation from animal research to clinical care has constantly seen huge disappointments. With the accumulation of detailed, information-rich data, human subjects start to contribute more to our understanding of pathobiology. It has been envisioned for some time that the combination of systems biology and epidemiology will be the prescription of personalized medicine [12]. The new developments in “blood systems biology” may be just enough to connect epidemiology, the “*n* > > 1” problem, to the realm of personalized medicine. That is, transcriptomics and metabolomics data from large cohorts can lead to robust models of risk factors and disease mechanisms. The future is bright also because biobank samples, even after long-term storage, can be still analyzed using newer technologies [102]. Close collaborations between computational scientists, epidemiologists and clinicians shall play a key role towards this future.
